# Prevalence of Trachoma in the Far North Region of Cameroon: Results of a Survey in 27 Health Districts

**DOI:** 10.1371/journal.pntd.0002240

**Published:** 2013-05-23

**Authors:** Blaise Noa Noatina, Giles Kagmeni, Marcellin Nimpa Mengouo, Henri Claude Moungui, Ann Tarini, Yaobi Zhang, Assumpta Lucienne Françoise Bella

**Affiliations:** 1 Programme National de Lutte Contre la Cécité, Ministère de la Santé, Yaoundé, Cameroun; 2 Faculté de Médecine et des Sciences Biomédicales, Université de Yaoundé I, Yaoundé, Cameroun; 3 World Health Organization, Yaoundé, Cameroun; 4 Délégation Régionale de la Santé Publique de l'Extrême-Nord, Maroua, Cameroun; 5 Helen Keller International, Yaoundé, Cameroun; 6 Helen Keller International, Regional Office for Africa, Dakar, Senegal; University of California San Diego School of Medicine, United States of America

## Abstract

**Background:**

Cameroon is known to be endemic with trachoma. To appreciate the burden of the disease and facilitate the national planning of trachoma control in the integrated control program for the neglected tropical diseases, an epidemiological mapping of trachoma was conducted in the Far North region in 2010–11.

**Methodology:**

A cross-sectional, cluster random sampling survey was carried out. The survey focused on two target populations: children aged 1 to 9 years for the prevalence of active trachoma and those aged 15 and over for the prevalence of trichiasis (TT). The sample frame was an exhaustive list of villages and neighborhoods of Health Districts (HDs). The World Health Organization simplified trachoma grading system was used for the recognition and registration of cases of trachoma.

**Principal Findings:**

48,844 children aged 1 to 9 years and 41,533 people aged 15 and over were examined. In children aged 1–9 years, the overall prevalence of trachomatous inflammation–follicular (TF) was 11.2% (95% confidence intervals (CI): 11.0–11.5%). More girls were affected than boys (p = 0.003). Thirteen (13) of 27 HDs in the region showed TF prevalence of ≥10%. The overall TT prevalence was 1.0% (95% CI: 0.9–1.1%). There were estimated 17193 (95% CI: 12576–25860) TT cases in the region. The prevalence of blindness was 0.04% (95% CI: 0.03–0.07%) and visual impairment was 0.09% (95% CI: 0.07–0.13%).

**Conclusions/Significance:**

The survey confirmed that trachoma is a public health problem in the Far North region with 13 HDs qualified for district-level mass drug administration with azithromycin. It provided a foundation for the national program to plan and implement the SAFE strategy in the region. Effort must be made to find resources to provide the surgical operations to the 17193 TT cases and prevent them from becoming blind.

## Author Summary

Trachoma is the leading infectious cause of blindness in the world, which is caused by repeated eye infections with the bacterium *Chlamydia trachomatis*. The global objective of trachoma control is to eliminate trachoma as a blinding disease worldwide by Year 2020, using the World Health Organization-endorsed SAFE strategy (Surgery to correct trichiasis, Antibiotics to treat infection, Facial cleanliness and Environmental improvement to interrupt transmission). In order to implement the control program, the knowledge of the disease distribution and prevalence in each district is essential. Disease mapping surveys were conducted in 27 health districts in the Far North region in Cameroon. Thirteen health districts have a prevalence of trachomatous inflammation–follicular ≥10% in children aged 1–9 years and qualify for district-level mass antibiotic treatment as well as intensive implementation of other components of SAFE. There are estimated to be 17193 trichiasis cases in the region, which need surgical operations to prevent from being blinded. The survey provided a foundation for the national program to plan and implement the SAFE strategy in the region.

## Introduction

Trachoma is the leading infectious cause of blindness in the world [1], [2]. It is one of the major neglected tropical diseases (NTDs) currently targeted by preventive chemotherapy [3], [4], and is one of the priority target diseases as part of the “Vision 2020: The Right to Sight” global initiative [5]. Trachoma is caused by ocular infections with *Chlamydia trachomatis*, which is transmitted from person to person through close contact, fomites or by eye seeking flies [6]. The chronic eye inflammation, trachomatous inflammation – follicular (TF) and trachomatous inflammation – intense (TI), produces scarring of the conjunctiva (trachomatous scarring, TS) that can subsequently cause trachomatous trichiasis (TT), resulting in inturned eyelashes. Continued abrasion of the cornea can subsequently lead to corneal opacity (CO), marked by a blurred pupil margin, severe visual impairment, and eventual blindness [7].

Trachoma is frequently found to cluster within endemic regions. Clustering has been demonstrated at the village, household and bedroom level [8]. This supports the hypothesis that transmission of *C. trachomatis* generally occurs with prolonged close contact between individuals. The signs of trachoma are strongly related to age, with TF and TI most commonly in preschool age children and TT typically in older adults (over 40 years of age) [9], [10]. Women are two to four times more likely to develop trichiasis than men [11], [12]. The disease is known to be highly correlated with poverty, lack of personal and community hygiene, limited access to health care and water. It is prevalent in Africa, Asia and some parts of Latin America, the Middle East and the Western Pacific [13]. It is estimated that 320 million people live in endemic areas and 8 million people suffer from TT [13]. Overall, Africa is the most affected continent. 27.8 million cases of active trachoma (68.5% of all) and 3.8 million cases of trichiasis (46.6% of all) are located in 28 of the 46 countries in the African Region, with an estimated population of 279 million living in endemic areas [14].

In 1997 the World Health Organization (WHO) established the Alliance for Global Elimination of Trachoma by the year 2020 (GET2020) [15]. It recommends an integrated SAFE strategy: Surgery to correct trichiasis, Antibiotics to treat infection, Facial cleanliness and Environmental improvement to interrupt transmission [16], [17]. The knowledge of the prevalence of trachoma at country and district level is essential for the planning and implementation of the interventions needed to eliminate this preventable cause of blindness and ultimately for the achievement of the elimination of trachoma as a blinding disease by year 2020. For antibiotic treatment, WHO recommends annual mass drug administration with azithromycin (Zithromax, donated by Pfizer Inc, USA) in districts where TF prevalence is ≥10% in children aged 1 to 9 years as well as intensive implementation of other components of SAFE. Similarly a prevalence of TT >1% is an indicator of a serious public health problem and the need for intensive efforts to provide high quality, accessible trichiasis surgical services [18].

In Cameroon, clinical observations confirmed the presence of trachoma in the Far North and North regions [19], but the magnitude of the problem had never been evaluated [20]. In 2006 an epidemiological survey was conducted in the Kolofata Health District (HD) in the Far North region. The results of this survey showed that trachoma is prevalent in this district, with TT and CO prevalence at 3.4% and 1.4% respectively in women aged 14 years and above, and TF prevalence in children aged 1 to 9 years at 21% [21]. Following this survey, mass treatment with azithromycin 1.5% eye drops was carried out in the Kolofata HD in 2008, 2009 and 2010 [22], [23], and Kolofata has now reached the goal of stopping mass treatment.

Since 2009, trachoma control in Cameroon has been integrated into the integrated national NTD control program, which is supported by the United States Department for International Development (USAID) NTD Control Program and currently the ENVISION Program managed by RTI International [24], [25]. To facilitate the planning of trachoma control activities in other HDs in the Far North region, an epidemiological mapping survey was conducted in 2010–11 in 27 HDs. The current paper presents the distribution of trachoma in the region and discusses the interventions needed to eliminate the blinding disease from the region.

## Methods

### Ethics statement

The survey was part of the national trachoma control program and was carried out by the National Blindness Control Program of the Ministry of Public Health according to the WHO endorsed survey methodology [26]. Prior to the survey, an ethical clearance was obtained from National Ethics Committee and an administrative authorization was obtained from the Ministry of Public Health. The Health District survey team visited villages, gave information about trachoma (causes, transmission, signs of the disease, how to prevent and treat), and explained the purpose of the survey. Verbal consent for the participation was sought from each participant aged 15 and above and for children aged 1 to 9 years consent was systematically sought from the family heads or guardians. Verbal consent was obtained because most people surveyed were illiterate. The National Ethics Committee and the Ministry of Public Health approved the use of oral consent. After ensuring that the potential subject has understood the information, the enumerator signed and dated the consent form and wrote his name and the family head's name on the form. The village head witnessed the all process. A copy of information notice was handed to those who could read French.

### Survey settings

The Far North Region is the northernmost constituent region of the Republic of Cameroon. It borders the North Region to the south, Chad to the east and Nigeria to the west. It is one of Cameroon's most culturally diverse regions. Over 50 different ethnic groups populate the area. The region is hot and dry with seasonal waterways. The climate is tropical and Sahelian, and the rainfall varies locally from 400 to 1500 mm per year. The population of the region was estimated at 3 480 414 inhabitants in 2010 [27], being the second most populated region in the country, and one of the most densely populated, 90.8 inhabitants/Km^2^ (4^th^ rank). 71.2% of the population lives in the rural area and 50.8% is less than 15 years. The region is divided into 28 health districts. The survey was done mainly in 2010 in 26 health districts and in 2011 in one district (Mora). Kolofata HD was surveyed in 2006 and the results from that survey were not included in the overall data analysis.

### Survey design and sampling

The survey was carried out using a cross-sectional two-stage random cluster sampling design according to the WHO recommendations [26]. The sample size for children aged 1–9 years for each district (n) was calculated using an expected TF prevalence of 20% (p) based on Kolofata survey data, a desired precision of estimate of 3.5% (m), an alpha risk of 5% (expressed as score = 1.96 (t)), and a design effect of 4 (g). An extra 5% was added to the sample size to adjust for non-respondents and other recording errors, giving rise to a sample of 2107 children aged 1–9 years per district, approximately 70 children per cluster.

For persons aged 15 and above, the sample size was calculated using an expected TT prevalence of 2.5% (p), a desired precision of estimate of 1% (m), an alpha risk of 5% (expressed as score = 1.96 (t)), and a design effect of 1.5 (g). An extra 5% was added to the sample size to adjust for non-respondents and other recording errors, giving rise to a sample size of 1475 per district, approximately 50 persons each cluster.

Within each HD, random cluster sampling was done in two stages. On the first stage, 30 clusters (20 clusters from four HDs due to the small number of villages) were randomly selected on the basis of probability proportional to population size and cumulative totals from a list of villages in the district. At the second stage, a selection of households was made within the clusters using the compact segment sampling method [28]. The villages were divided into segments that contained approximately the same number of houses. Segments were then randomly selected until enough numbers of children aged 1 to 9 years and those aged 15 and above were reached.

### Household survey

Within each household, all eligible household residents (who have resided at least six months in the village or neighborhood at the time of survey) including those absent at the time of visit were identified and enumerated for examination. Those household members present at the time of survey and willing to participate in the survey were examined.

The WHO simplified trachoma grading system was used for the recognition and registration of cases of trachoma [29], [30]. Ophthalmic nurses or ophthalmic medical assistants, some of whom participated in the Kolofata survey in 2006, were selected for training as trachoma graders. They were trained for 4 days. The first three days were classroom training on standardized procedures for selection of households, enumeration, examination, identification of trachoma, and collecting and managing data. For the recognition of signs of trachoma, slides showing pictures of various forms of trachoma were used. They were trained on reliably recognizing TF, TT and CO signs using these pictures and on safely examining eyes of adults, children and infants. The training also included discussions on how to prevent transmission of eye infections from one person to another during the process of examination and on how to recognize the clinical signs of other national priority eye diseases (i.e. cataract, glaucoma, and refractive errors) and how to refer or treat such patients. At the end of classroom training, the trainees were projected 100 pictures showing various signs of trachoma and were tested in their ability to correctly identify the trachoma grades on an answer paper. Those who had 90% of correct answers were finally recruited as trachoma graders in the team. The training sessions in the field on the fourth day included household selection and enumeration only.

The eyelids and cornea were first examined for deviated lashes and/or corneal opacities. When such signs were found, visual acuity was measured using Snellen E optotypes. If there was visual loss, trachoma accountability was sought (presence of conjunctival TS, TT and/or pannus). The upper eyelids were routinely everted and examined using a binocular loupe (magnification 2.5), under good light conditions. Everyone included in the study were reviewed by a senior Ophthalmologist.

The diagnosis of both eyes was recorded on the data collection form. The examiner ensured that the data collection form was completed in accordance to protocol requirements before proceeding to the next person. In cases that different grades of trachoma were observed between both eyes, the more serious condition was used for the individual. The blindness and low vision were classified according to the International Classification of Diseases [31].

All those who showed signs of active trachoma were treated locally with 1% tetracycline ointment. TT cases were referred to an ophthalmologic center for free surgical intervention.

### Data management and analysis

Data were entered into database using the EpiInfo 3.5.1 software and transferred to SPSS version 19 (IBM) for analysis. Data were cleaned, and about 1.3% of the total entries had missing or invalid data, particularly on age and therefore was not included in the final analysis. When calculating the overall prevalence in the region or prevalence by sex, samples were weighted according to the proportion of population in each district among the total population in 2010 in the region projected from the 2005 national census with an annual growth rate of 2.6% [27], and the SPSS Complex Samples module was used with the districts as strata and villages as clusters. The 95% confidence intervals (CIs) of the prevalence were calculated with the Wilson score method without continuity correction [32], using the CI calculator (available: http://vl.academicdirect.org/applied_statistics/binomial_distribution/ref/CIcalculator.xls). Differences in prevalence of trachoma were compared using the Chi-squared test. Geographical prevalence maps were drawn using ArcGIS version 10 (ESRI, Inc). Trichiasis cases in each district were estimated according to the population in 2010 and the overall TT prevalence in each district.

## Results

### Study population

Throughout 27 health districts surveyed, a total of 51,028 children aged 1–9 years were enumerated from the selected households, and 48,844 were examined, a participation rate of 95.7%. A total of 46,255 people aged 15 and over were identified and 41,533 were examined, representing a participation rate of 89.8%. Demographic data of the surveyed population in each district are shown in Table 1.

**Table 1:** Demographic data of the surveyed population in each health district.

District	Estimated population in 2010^a^	No of clusters surveyed	No of children (1–9 years) enumerated	No of children (1–9 years) examined	Participation rate (%)	Proportion of females (%)	No of people (≥15 years) enumerated	No of people (≥15 years) examined	Participation rate (%)	Proportion of females (%)
Bogo	94,161	30	1292	1272	98.5	47.6	1253	1228	98.0	48.7
Bourha	71,947	30	1964	1936	98.6	46.0	1493	1466	98.2	53.8
Goulfey	72,866	30	1587	1509	95.1	49.4	1810	1447	79.9	46.6
Guere	115,811	30	1905	1730	90.8	48.2	1423	1188	83.5	57.6
Guidiguis	163,279	30	2307	2299	99.7	51.4	1796	1795	99.9	54.4
Hina	100,661	30	2108	2083	98.8	50.9	1586	1579	99.6	58.5
Kaele	116,351	30	2051	2028	98.9	49.0	2609	2569	98.5	52.8
Kar Hay	117,755	30	1694	1693	99.9	50.4	1373	1373	100.0	55.1
Kousseri	180,791	30	2079	2000	96.2	49.4	1874	1766	94.2	50.0
Koza	169,081	30	1872	1603	85.6	48.5	2326	1784	76.7	55.3
Mada	111,170	30	2607	2088	80.1	45.9	2299	1097	47.7	49.9
Maga	169,040	30	2118	2103	99.3	48.6	1762	1758	99.8	60.1
Makary	127,355	30	1808	1795	99.3	40.6	1474	1452	98.5	42.8
Maroua-Rural	226,922	30	1896	1786	94.2	48.0	1520	1364	89.7	53.7
Maroua-Urban	241,451	30	1934	1815	93.8	51.8	2138	1749	81.8	52.7
Meri	198,420	30	2236	2207	98.7	47.9	2334	2247	96.3	54.7
Mindif	65,099	30	669	639	95.5	52.0	999	959	96.0	50.9
Mogode	102,809	30	2138	2073	97.0	50.1	1729	1514	87.6	55.6
Mokolo	208,876	30	2447	2305	94.2	49.7	2103	1746	83.0	50.1
Mora	167,269	30	1980	1980	100.0	46.9	1759	1759	100.0	58.2
Moulvoudaye	98,793	20	994	940	94.6	47.8	1280	1215	94.9	50.0
Moutourwa	42,270	20	1895	1835	96.8	48.3	1373	1283	93.4	52.7
Pette	49,671	20	2230	2216	99.4	49.6	1783	1726	96.8	56.7
Roua	90,460	20	2127	2013	94.6	49.5	2241	1982	88.4	54.6
Tokombere	136,615	30	1923	1817	94.5	49.9	1629	1463	89.8	53.5
Vele	78,945	20	1637	1637	100.0	47.2	1105	1030	93.2	53.2
Yagoua	228,273	30	1530	1442	94.2	49.8	1184	994	84.0	59.0
**Total**	**3,546,141**		**51,028**	**48,844**	**95.7**	**48.7**	**46,255**	**41,533**	**89.8**	**53.5**

a Estimated according to the national census data [27].

In total, 97,283 persons were identified and 90,377 were examined, an overall participation rate of 92.9% (see Table 1). Participation rate ranged from 47.7% (Mada) to 100% (Kar Hay) for persons aged 15 and older and 80.1% to 100% for children aged 1 to 9 years.

### Prevalence of active trachoma in children aged 1–9 years

**Figure 1:**
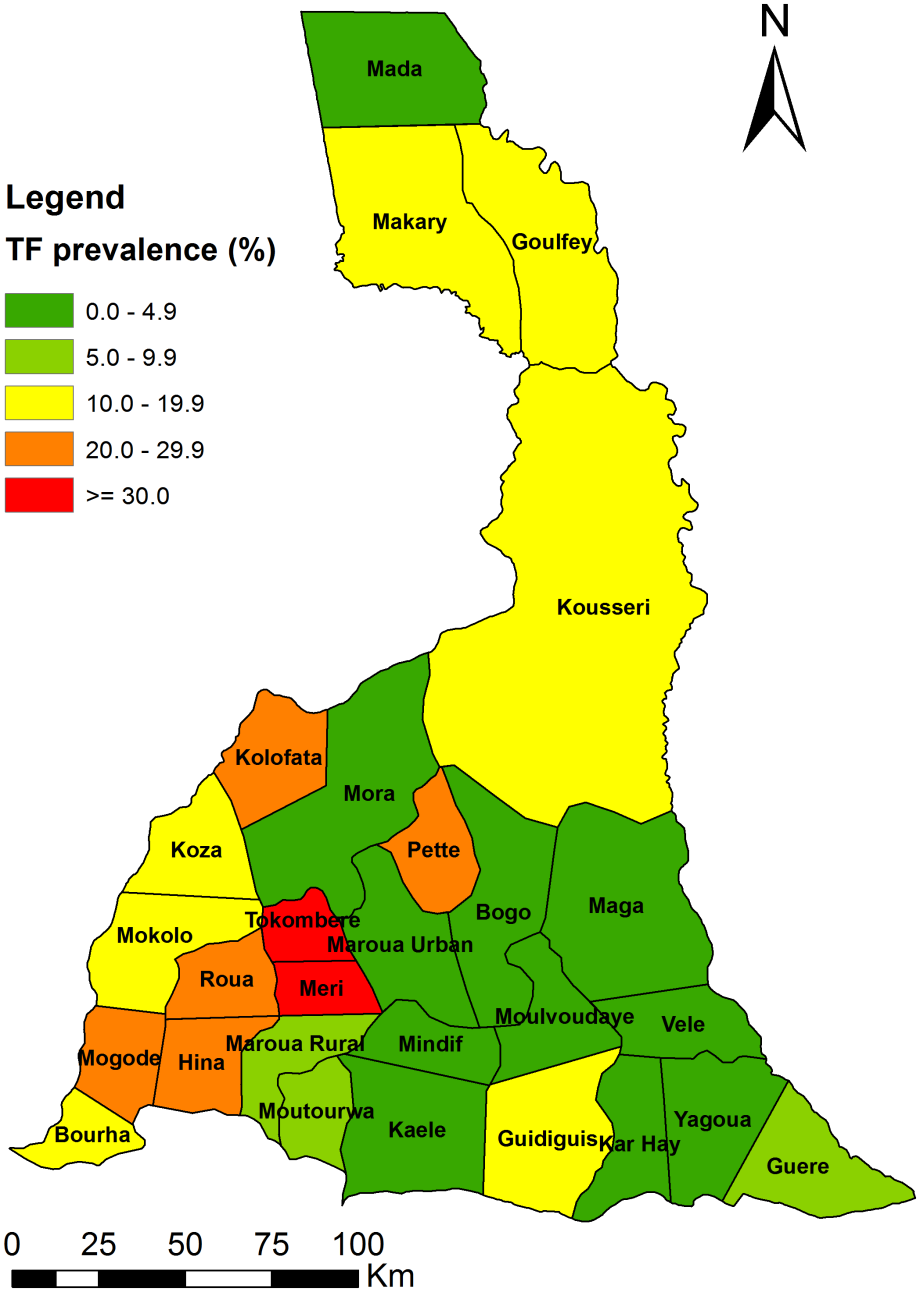
Distribution of TF prevalence in children aged 1–9 years in the Far North region.

Table 2 summarizes the prevalence of trachoma in the Far North region by district. The overall prevalence of TF is 11.2% (95% CI: 11.0–11.5%) in the 27 districts surveyed in the region. The prevalence ranged from 0% to 42.5% by district. Thirteen (13) of 27 health districts surveyed, in the region had TF prevalence ≥10% (Figure 1). These are Bourha, Goulfey, Guidiguis, Hina, Kousseri, Koza, Makari, Meri, Mogode, Mokolo, Pette, Roua, and Tokombere HDs.

**Table 2:** Prevalence (%) of trachoma and estimated TT cases in the Far North Region.

District	No of children aged 1–9 years examined	TF in children aged 1–9 years (95% CI)	Active trachoma (TF&TI) in children aged 1–9 years (95% CI)	No of people ≥15 years examined	TT in people ≥15 years (95% CI)	TT in all ages (95% CI)	Estimated TT cases (95% CI)
Bogo	1273	0.0 (0.0–0.3)	0.0 (0.0–0.3)	1228	0.1 (0.0–0.5)	0.04 (0.01–0.23)	38 (9–217)
Bourha*	1936	10.1 (8.9–11.6)	11.0 (9.6–12.4)	1466	0.2 (0.1–0.6)	0.09 (0.03–0.26)	63 (22–187)
Goulfey*	1509	11.1 (9.6–12.8)	11.1 (9.6–12.8)	1449	2.8 (2.0–3.7)	1.39 (1.02–1.88)	1011 (743–1370)
Guere	1730	7.5 (6.4–8.9)	7.6 (6.4–8.9)	1188	1.2 (0.7–2.0)	0.48 (0.29–0.80)	556 (336–926)
Guidiguis*	2299	16.9 (15.4–18.5)	17.7 (16.2–19.3)	1795	0.0 (0.0–0.2)	0 (0.00–0.09)	0 (0–147)
Hina*	2083	21.6 (19.9–23.4)	21.7 (20.0–23.5)	1579	0.2 (0.1–0.6)	0.08 (0.03–0.24)	82 (30–242)
Kaele	2028	1.2 (0.8–1.8)	1.3 (0.9–1.9)	2565	0.0 (0.0–0.2)	0 (0.00–0.08)	0 (0–93)
Kar Hay	1694	0.5 (0.2–0.9)	0.5 (0.2–0.9)	1373	1.0 (0.6–1.7)	0.46 (0.27–0.77)	538 (318–907)
Kousseri*	2003	12.5 (11.1–14.0)	13.3 (11.9–14.9)	1766	0.6 (0.4–1.1)	0.42 (0.26–0.69)	768 (470–1247)
Koza*	1603	10.7 (9.3–12.3)	20.5 (18.6–22.6)	1783	0.1 (0.0–0.4)	0.06 (0.02–0.22)	100 (34–372)
Mada	2089	1.8 (1.3–2.5)	1.8 (1.3–2.5)	1096	0.7 (0.4–1.4)	0.25 (0.13–0.49)	279 (145–545)
Maga	2104	1.8 (1.3–2.4)	1.8 (1.3–2.4)	1758	0.1 (0.0–0.3)	0.03 (0.00–0.15)	44 (0–254)
Makary*	1795	14.2 (12.6–15.8)	14.8 (13.2–16.5)	1450	4.1 (3.2–5.2)	1.85 (1.44–2.37)	2353 (1834–3018)
Maroua-Rural	1787	8.7 (7.5–10.1)	8.8 (7.6–10.2)	1364	0.0 (0.0–0.3)	0 (0.00–0.12)	0 (0–272)
Maroua-Urban	1815	4.4 (3.5–5.4)	4.7 (3.9–5.8)	1748	0.0 (0.0–0.2)	0.03 (0.00–0.16)	68 (0–386)
Meri*	2207	33.1 (31.1–35.1)	33.1 (31.1–35.1)	2247	1.5 (1.1–2.1)	0.76 (0.55–1.06)	1515 (1091–2103)
Mindif	640	0.0 (0.0–0.6)	0.0 (0.0–0.6)	959	0.0 (0.0–0.4)	0 (0.00–0.24)	0 (0–156)
Mogode*	2073	20.0 (18.3–21.8)	32.2 (30.3–34.3)	1514	7.3 (6.1–8.7)	3.12 (2.60–3.74)	3210 (2673–3845)
Mokolo*	2305	13.1 (11.7–14.5)	18.0 (16.5–19.7)	1746	5.3 (4.4–6.5)	2.30 (1.88–2.80)	4795 (3927–5849)
Mora	1980	3.8 (3.0–4.7)	4.3 (3.5–5.3)	1759	0.1 (0.0–0.3)	0.05 (0.01–0.19)	89 (17–318)
Moulvoudaye	940	0.0 (0.0–0.4)	0.0 (0.0–0.4)	1215	0.0 (0.0–0.3)	0 (0.00–0.18)	0 (0–178)
Moutourwa	1836	6.3 (5.3–7.5)	6.6 (5.6–7.8)	1282	0.0 (0.0–0.3)	0 (0.00–0.12)	0 (0–51)
Pette*	2216	23.1 (21.4–24.9)	23.1 (21.4–25.0)	1726	0.7 (0.4–1.2)	0.30 (0.17–0.53)	151 (84–263)
Roua*	2014	25.5 (23.7–27.5)	40.1 (38.0–42.3)	1982	0.1 (0.0–0.3)	0.03 (0.00–0.14)	23 (0–127)
Tokombere*	1817	42.5 (40.2–44.8)	42.6 (40.3–44.9)	1463	1.1 (0.7–1.8)	0.49 (0.30–0.79)	666 (410–1079)
Vele	1637	2.7 (2.0–3.6)	3.2 (2.4–4.1)	1030	0.0 (0.0–0.4)	0 (0.00–0.14)	0 (0–111)
Yagoua	1442	4.9 (3.9–6.2)	5.0 (4.0–6.2)	994	0.9 (0.5–1.7)	0.37 (0.19–0.70)	843 (434–1598)
**Total**	**48,844**	**11.2 (11.0–11.5)**	**12.9 (12.6–13.2)**	**41,533**	**1.0 (0.9–1.1)**	**0.49 (0.44–0.53)**	**17193 (12576–25860)**

* HDs marked with a (*) qualify for district level MDA with azithromycin.

The rate of TF was higher in girls than in boys (11.7% vs 10.8% p = 0.003).

### TT in people aged 15 and older, visual impairment and blindness due to trachoma

**Figure 2:**
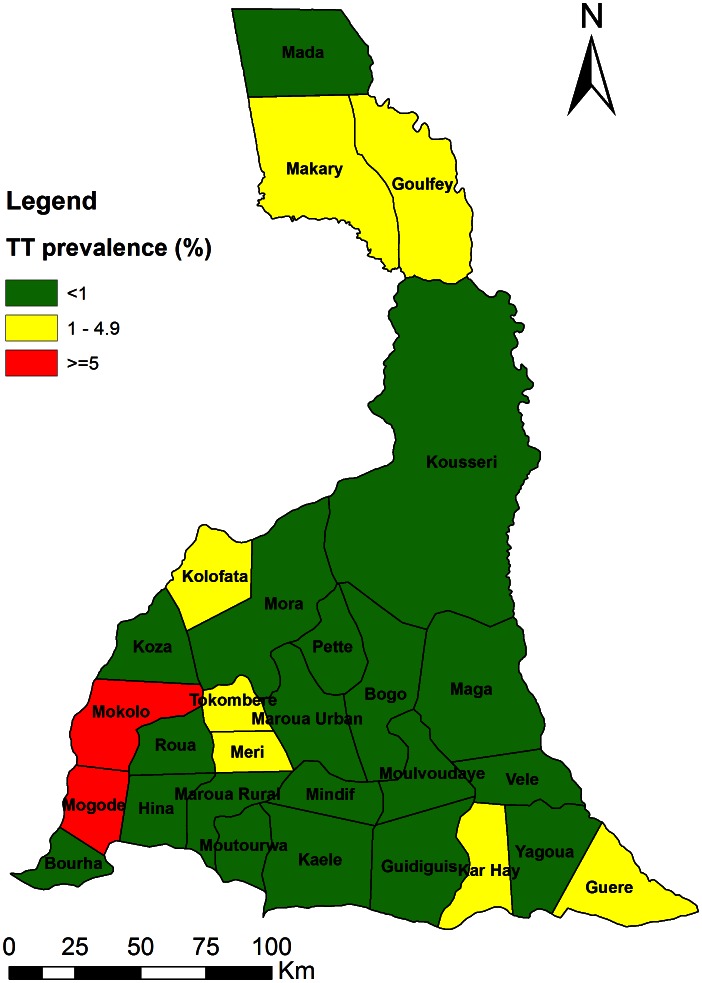
Distribution of TT prevalence in adults aged 15 years and over in the Far North region.

The prevalence of TT in individuals aged 15 and older is 1.02% (95% CI: 0.93–1.13%) in 27 districts surveyed in the region. The prevalence ranged from 0%–7.3%. Eight (8) health districts in the region had a prevalence of TT ≥1%: Goulfey, Guere, Kar Hay, Makari, Meri, Mogode, Mokolo and Tokombere HDs (Figure 2).

The prevalence of TT in women and men was 1.13% (95% CI: 1.00–1.27%) and 0.91% (95% CI: 0.78–1.05%) respectively, which are not significantly different (p = 0.076). The prevalence of CO, impaired vision and blindness among individuals aged 15 and older were 0.59% (95% CI: 0.52–0.67%), 0.09% (95% CI: 0.07–0.13%), 0.04% (95% CI: 0.03–0.07%) respectively. There is no significant difference between women and men in any of these (p>0.05), however there is a significant trend of increasing prevalence with age (Table 3).

**Table 3:** Prevalence (%) of TT, CO, impaired vision and blindness due to trachoma in people of ≥15 years.

	TT (95% CI)	CO % (95% CI)	Impaired vision % (95% CI)	Blindness % (95% CI)
***By age group***				
15–24	0.08 (0.05–0.15)	0.07 (0.04–0.13)	0 (-)	0 (-)
25–34	0.19 (0.12–0.30)	0.09 (0.04–0.16)	0 (-)	0 (-)
35–44	0.57 (0.42–0.79)	0.38 (0.26–0.56)	0.03 (0.01–0.11)	0 (-)
45–54	1.67 (1.34–2.10)	1.14 (0.86–1.49)	0.07 (0.02–0.20)	0.09 (0.04–0.23)
55–64	3.07 (2.50–3.79)	1.75 (1.33–2.31)	0.30 (0.17–0.61)	0.24 (0.12–0.52)
65–74	6.65 (5.59–7.95)	3.08 (2.36–4.00)	0.75 (0.44–1.29)	0.07 (0.01–0.33)
75 et +	9.24 (7.52–11.34)	5.52 (4.21–7.24)	1.30 (0.69–2.21)	0.68 (0.31–1.47)
***By sex***				
Male	0.91 (0.78–1.05)	0.59 (0.49–0.71)	0.12 (0.08–0.17)	0.05 (0.02–0.09)
Female	1.13 (1.00–1.27)	0.60 (0.50–0.71)	0.07 (0.04–0.12)	0.04 (0.02–0.08)
**Total**	**1.02 (0.93–1.13)**	**0.59 (0.52–0.67)**	**0.09 (0.07–0.13)**	**0.04 (0.03–0.07)**

### Estimation of trichiasis cases in the region

Overall prevalence of TT among all ages examined was shown in Table 2, with an overall prevalence of 0.49% (95% CI: 0.44–0.53%). The number of trichiasis cases in each district was estimated using the overall TT prevalence in each district. There are estimated 17, 193 cases of trichiasis in the region with 95% CI of 12, 576 to 25,860 (Table 2). Most of the cases are concentrated in five HDs, representing 75% of the total cases in the surveyed HDs. These HDs are Mokolo (4,795), Mogode (3,210), Makary (2,353), Meri (1,515) and Goulfey (1,011).

## Discussion

Knowledge of trachoma prevalence at district level is particularly important in planning the national trachoma control program in order to achieve the goal of eliminating trachoma as a blinding disease by year 2020. District level mapping of trachoma prevalence was carried out in the Far North region in Cameroon. With a prevalence of trachoma (TF) of 11.2%, and TT of 1.02%, the Far North region is indeed an endemic area for trachoma. The prevalence of TF is highly variable, ranging from 0% (Bogo, Mindif, Moulvoudaye HDs) to 42.6% (Tokombere HD). It is known that the prevalence of trachoma may vary from one region to another, from one district to another and even from one community to another [13]. Following the 2006 survey in Kolofata HD, where three rounds of mass treatment with azithromycin 1.5% eye drops had been carried out [22], [23], already reaching the goal of stopping mass treatment, this work has identified additional 13 HDs in which the prevalence of TF in the 1–9 years is ≥10%, qualifying for district level mass drug administration with azithromycin (Figure 1). A total population of 1.67 million in the 13 HDs needs to be targeted each year for treatment for at least three years.

The HDs with prevalence of ≥10% are located mainly along the mountain range near the border with Nigeria (Monts Mandara). Risk factors for infection with trachoma include crowding and household clustering; insufficient access to water, poor sanitation and facial hygiene, and young children as reservoir of infection [33]. In the current survey, HDs with high prevalence of active trachoma in the mountains bordering Nigeria are characterized by limited access to water. The fact that high prevalence of active trachoma in the Far North region is distributed mainly in HDs near international borders highlights the need of cross-border collaboration on timing of interventions as for other NTDs. Otherwise the effect of mass antibiotic treatment may be considerably reduced by population movement and reinfection.

Some HDs have very high active trachoma prevalence, comparable to those found in Mali with a prevalence of active trachoma ranging from 23 to 47% [34], in Chad, a neighboring country, with TF prevalence of 31.5% [35], and in Niger with the prevalence of active trachoma ranging from 3 to 82% [36]. The distribution of trachoma signs by gender shows that active trachoma (including TF and TI) is slightly more common among girls (13.5%) than boys (12.4%). Though statistically significant, it is unlikely to be programmatically different, as the mass drug administration program would still be keen to ensure high levels of antibiotic treatment coverage in both girls and boys.

The overall prevalence of TT in the 27 HDs surveyed in the region of the Far North in people aged 15 and over is 1.02%, ranging from 0% to 7.3%. Eight HDs had TT prevalence of ≥1%, which is one of the thresholds, along with TF prevalence of ≥10% in children aged 1–9 years, set by the WHO for implementing trachoma control activities. Additionally, according to the 2006 survey, Kolofata HD had a TT prevalence of 3.4% (95% CI: 2.4–4.7%) in women aged 14 and over [21]. TT prevalence increases with age and the peak is reached in the 75+ age group as well in women as in men. TT prevalence in women (1.13%) was statistically similar to that found in men (0.91%). This does not seem to be in line with reports that women were two to four times more likely to have trichiasis than men [12]. From the survey data, taking into account the TT cases in Kolofata HD (2,929–5,737), we estimate that there are 15,506 to 31,597 people suffering from TT in the region, who are at the risk of going blind and need surgical corrections.

The study had a number of limitations. Firstly, overall participation in the survey was very high, 95% for the sample of children aged 1–9 years and 89.1% for the population aged 15 years and over. However, it is noted that in Mada HD the participation among people aged 15 years and over was particularly low (47.7%). Although this may have affected the estimation of TT cases in the HD, it does not affect the classification of the endemic status of this HD based on the TF prevalence in children aged 1–9 years with an 80% participation rate, and it also does not affect the program decisions on other HDs. Several factors may explain the low participation in the district: the nomadic populations, the coincidence with the holding of a livestock sale market days, and the inaccessibility of the village because of floods of relief. Secondly, for the training of surveyors, we used pictures showing the various forms of trachoma instead of trachoma patients for the recognition of trachoma grading. These may have affected the training quality in grading trachoma. However, only surveyors who obtained at least 90% of answers in correctly grading trachoma during training sessions were finally recruited in the survey team. Therefore, the overall quality of this survey should not have been affected. Thirdly, this survey did not involve environmental and socio economic risk factors for trachoma occurrence. The emphasis was to determine trachoma prevalence for planning and implementing the interventions needed to eliminate trachoma in the region. Therefore it was not possible to make a correlation between our findings and trachoma risk factors. However, it is well known that trachoma is highly correlated with poverty, lack of personal and community hygiene, limited access to health care and water [20].

The mapping survey has filled the knowledge gap in trachoma distribution in the Far North region. It has enabled the Ministry of Public Health to assess the magnitude and the distribution of the disease in the region to design a national plan for elimination of trachoma. A national strategic plan for trachoma control has recently been finalized. In the Far North region, the four components of the SAFE strategy will be implemented in 13 HDs with TF prevalence of ≥10%: Bourha, Goulfey, Guidiguis, Hina, Kousseri, Koza, Makari, Meri, Mogode, Mokolo, Pette, Roua, and Tokombere. Mass drug administration with azithromycin started in 2011 in 8 HDs: Bourha, Hina, Koza, Meri, Mogode, Pette, Roua, Tokombere. In 2012 five more HDs shall be involved in mass drug administration with azithromycin: Goulfey, Guidiguis, Koza, Kousseri, Makari. In those HDs with large estimated number of TT cases, particularly in Goulfey, Kolofata, Makary, Meri, Mogode and Mokolo, effort must be made to provide surgical operations to the TT cases to prevent them from becoming blind. Given the limited funds for TT surgery in the current NTD funding, the national program needs to find the funding sources for such services.

## Supporting Information

Checklist S1 STROBE Checklist.
(DOC)

## References

[1] Resnikoff S, Pascolini D, Etya'ale D, Kocur I, Pararajasegaram R, et al (2004) Global data on visual impairment in the year 2002. Bull World Health Organ 82: 844–851. 15640920 PMC2623053

[2] West S, Sommer A (2001) Prevention of blindness and priorities for the future. Bull World Health Organ 79: 244–248. 11285670 PMC2566384

[3] Hotez PJ, Kamath A (2009) Neglected tropical diseases in sub-saharan Africa: review of their prevalence, distribution, and disease burden. PLoS Negl Trop Dis 3: e412. 19707588 10.1371/journal.pntd.0000412 PMC2727001

[4] Reddy M, Gill SS, Kalkar SR, Wu W, Anderson PJ, et al (2007) Oral drug therapy for multiple neglected tropical diseases: a systematic review. JAMA 298: 1911–1924. 17954542 10.1001/jama.298.16.1911

[5] McGavin DD (1999) The global initiative for the elimination of avoidable blindness - vision 2020: the right to sight. Community Eye Health 12: 32. 17491991 PMC1706008

[6] Jones BR (1975) The prevention of blindness from trachoma. Trans Ophthalmol Soc U K 95: 16–33. 775692

[7] Bowman RJ, Jatta B, Cham B, Bailey RL, Faal H, et al (2001) Natural history of trachomatous scarring in The Gambia: results of a 12-year longitudinal follow-up. Ophthalmology 108: 2219–2224. 11733262 10.1016/s0161-6420(01)00645-5

[8] Bailey R, Osmond C, Mabey DC, Whittle HC, Ward ME (1989) Analysis of the household distribution of trachoma in a Gambian village using a Monte Carlo simulation procedure. Int J Epidemiol 18: 944–951. 2621031 10.1093/ije/18.4.944

[9] West SK, Munoz B, Turner VM, Mmbaga BB, Taylor HR (1991) The epidemiology of trachoma in central Tanzania. Int J Epidemiol 20: 1088–1092. 1800408 10.1093/ije/20.4.1088

[10] Dawson CR, Daghfous T, Messadi M, Hoshiwara I, Schachter J (1976) Severe endemic trachoma in Tunisia. Br J Ophthalmol 60: 245–252. 1276112 10.1136/bjo.60.4.245 PMC1017485

[11] Courtright P, West SK (2004) Contribution of sex-linked biology and gender roles to disparities with trachoma. Emerg Infect Dis 10: 2012–2016. 15550216 10.3201/eid1011.040353 PMC3328994

[12] West S, Nguyen MP, Mkocha H, Holdsworth G, Ngirwamungu E, et al (2004) Gender equity and trichiasis surgery in the Vietnam and Tanzania national trachoma control programmes. Br J Ophthalmol 88: 1368–1371. 15489474 10.1136/bjo.2004.041657 PMC1772400

[13] Haddad D (2012) The end game for blinding trachoma. World Ophthalmology News.

[14] Mariotti SP, Pascolini D, Rose-Nussbaumer J (2009) Trachoma: global magnitude of a preventable cause of blindness. Br J Ophthalmol 93: 563–568. 19098034 10.1136/bjo.2008.148494

[15] WHO (1997) Planning for the global elimination of trachoma (GET): report of a WHO consultation. Geneva: World Health Organization.

[16] Mariotti SP, Prüss A (2001) The SAFE strategy: Preventing trachoma - a guide for environmental sanitation and improved hygiene. Geneva: World Health Organization.

[17] Emerson PM, Lindsay SW, Walraven GE, Faal H, Bogh C, et al (1999) Effect of fly control on trachoma and diarrhoea. Lancet 353: 1401–1403. 10227221 10.1016/S0140-6736(98)09158-2

[18] WHO (2003) Report of the 2nd Global Scientific Meeting on trachoma. Geneva: World Health Organization.

[19] OSF Rapport d'activités Ophtalmo Sans Frontières au Cameroun 1987–2007. Ophtalmo sans Frontières.

[20] Polack S, Brooker S, Kuper H, Mariotti S, Mabey D, et al (2005) Mapping the global distribution of trachoma. Bull World Health Organ 83: 913–919. 16462983 PMC2626493

[21] Bensaïd P, Huguet P, Goldschmidt P, Einterz E (2007) Le trachome au Cameroun : résultats d'une enquête épidémiologique dans le district de Kolofata dans la province de l'Extrême-Nord. Rev int Trach Pathol Ocul Trop et Subtrop Santé Publique 84: 79–103.

[22] Huguet P, Bella L, Einterz EM, Goldschmidt P, Bensaid P (2010) Mass treatment of trachoma with azithromycin 1.5% eye drops in the Republic of Cameroon: feasibility, tolerance and effectiveness. Br J Ophthalmol 94: 157–160. 19692356 10.1136/bjo.2009.161513 PMC2922718

[23] Amza A, Goldschmidt P, Einterz E, Huguet P, Olmiere C, et al (2010) Elimination of active trachoma after two topical mass treatments with azithromycin 1.5% eye drops. PLoS Negl Trop Dis 4: e895. 21124889 10.1371/journal.pntd.0000895 PMC2990706

[24] Linehan M, Hanson C, Weaver A, Baker M, Kabore A, et al (2011) Integrated implementation of programs targeting neglected tropical diseases through preventive chemotherapy: proving the feasibility at national scale. Am J Trop Med Hyg 84: 5–14. 21212194 10.4269/ajtmh.2011.10-0411 PMC3005506

[25] Hanson C, Weaver A, Zoerhoff KL, Kabore A, Linehan M, et al (2012) Integrated implementation of programs targeting neglected tropical diseases through preventive chemotherapy: identifying best practices to roll out programs at national scale. Am J Trop Med Hyg 86: 508–513. 22403327 10.4269/ajtmh.2012.11-0589 PMC3284372

[26] Ngondi J, Reacher M, Matthews F, Brayne C, Emerson P (2009) Trachoma survey methods: a literature review. Bull World Health Organ 87: 143–151. 19274367 10.2471/BLT.07.046326 PMC2636192

[27] BUCREP (2010) 3e recensement général de la population et de l'habitat : La population du Cameroun en 2010. Bureau Central des Recensements et des Etudes de Population, Yaoundé, Cameroon.

[28] Kuper H, Polack S, Limburg H (2006) Rapid assessment of avoidable blindness. Community Eye Health 19: 68–69. 17515970 PMC1871676

[29] Taylor HR, West SK, Katala S, Foster A (1987) Trachoma: evaluation of a new grading scheme in the United Republic of Tanzania. Bull World Health Organ 65: 485–488. 3500801 PMC2491034

[30] Thylefors B, Dawson CR, Jones BR, West SK, Taylor HR (1987) A simple system for the assessment of trachoma and its complications. Bull World Health Organ 65: 477–483. 3500800 PMC2491032

[31] WHO (2010) International classification of diseases. World Health Organization.

[32] Newcombe RG (1998) Two-sided confidence intervals for the single proportion: comparison of seven methods. Stat Med 17: 857–872. 9595616 10.1002/(sici)1097-0258(19980430)17:8<857::aid-sim777>3.0.co;2-e

[33] Wright HR, Turner A, Taylor HR (2008) Trachoma. Lancet 371: 1945–1954. 18539226 10.1016/S0140-6736(08)60836-3

[34] Schemann JF, Sacko D, Banou A, Bamani S, Bore B, et al (1998) Cartography of trachoma in Mali: results of a national survey. Bull World Health Organ 76: 599–606. 10191556 PMC2312488

[35] Madani MO, Huguet P, Mariotti SP, Dezoumbe D, Tosi C, et al (2003) Trachoma in Chad: results of an epidemiological survey. Sante 13: 9–15. 12925317

[36] Abdou A, Nassirou B, Kadri B, Moussa F, Munoz BE, et al (2007) Prevalence and risk factors for trachoma and ocular Chlamydia trachomatis infection in Niger. Br J Ophthalmol 91: 13–17. 16899525 10.1136/bjo.2006.099507 PMC1857562

